# Thermodynamic analysis of volatile organometallic fission products

**DOI:** 10.1007/s10967-015-4653-9

**Published:** 2015-12-17

**Authors:** John D. Auxier, Jacob A. Jordan, S. Adam Stratz, Shayan Shahbazi, Daniel E. Hanson, Derek Cressy, Howard L. Hall

**Affiliations:** Department of Nuclear Engineering, University of Tennessee, 301 Middle Dr., Pasqua Nuclear Engineering Building, Knoxville, TN 37996 USA; Radiochemistry Center of Excellence (RCOE), University of Tennessee, 1508 Middle Dr., Ferris Hall, Knoxville, TN 37996 USA; Center for Public Policy, Institute for Nuclear Security, University of Tennessee, 1640 Cumberland Ave. Howard Baker Jr, Knoxville, TN 37996 USA; Department of Chemistry, University of Tennessee, 552 Circle Dr., Buehler Hall, Knoxville, TN 37996 USA

**Keywords:** Nuclear forensics, Thermogravimetric analysis, Differential thermal analysis, Rapid separations

## Abstract

The ability to perform rapid separations in a post nuclear weapon detonation scenario is an important aspect of national security. In the past, separations of fission products have been performed using solvent extraction, precipitation, etc. The focus of this work is to explore the feasibility of using thermochromatography, a technique largely employed in superheavy element chemistry, to expedite the separation of fission products from fuel components. A series of fission product complexes were synthesized and the thermodynamic parameters were measured using TGA/DSC methods. Once measured, these parameters were used to predict their retention times using thermochromatography.

## Introduction

The illicit use of nuclear material is one of the major challenges of the modern era. The threat of nuclear proliferation and nuclear terrorism is a continued and growing concern [1–5]. This threat has been recognized by Congress and was the primary motivation for the passage of the Nuclear Forensics and Attribution Act in 2010 [6]. This act called for the development of a credible capability for identifying sources of nuclear material used in an act of terrorism, and also acknowledged the challenge presented by the dwindling number of radiochemical programs and facilities in the United States. In an effort to improve the nuclear forensics capability, this work seeks to develop a method to reduce required to perform separation and detection of fission products found in a post-detonation scenario.

To address this need, this work will highlight the efforts to utilize thermodynamic measurements to allow the development of a technique known as thermo-chromatography [2, 3]. Thermochromatography, or thermally driven physiochemical separation, is a technique that has been used almost exclusively in the superheavy element community due to its capability to utilize the volatile nature of carbonyl complexes giving rise to rapid separation times [7, 8]. The focus of this work is to expand this application to new elements, in particular the rare earth elements, since they comprise the heavy end of the fission product curve. As such they can be utilized to identify a variety of nuclear forensic signatures based on their elemental presence and isotopic ratios.

In order to separate these fission products using thermochromatography, they must be volatile at a temperature that is attainable by the GCMS instrument. As oxides or chlorides, the fission product complexes simply non-volatile at the maximum operating temperature of a GCMS. To address this issue, a highly volatile ligand was attached to the fission products to allow for volatility at temperatures attainable by the GCMS instrument. A β-diketonate ligand was chosen for this process due to its high volatility and ease of synthesis.

The present work serves to approximate the thermodynamics, specifically the sublimation enthalpy, of various organometallic complexes comprised of rare earth metals with ligand. From this, the adsorption enthalpy can be empirically deduced. These thermodynamic quantities are essential in understanding the relative separations on a column. Such relationships can estimate retention times on a chromatography column. The goal of present and future work is to provide rapid separations of these common fission product compounds through experiment and simulation.

## Theory

Thermochromatography is the separation of compounds via thermal mechanisms, either through thermal gradients or isothermal environments. Understanding the thermal characteristics of the compound is therefore necessary, with adsorption enthalpy being an important parameter when discussing column chromatography. Empirical correlations between the adsorption and sublimation enthalpy have been found for various heavy metal oxides by Eichler et al. [7] As part of this effort, the thermodynamic parameters, Δ*G*_sub_, Δ*S*_sub_, Δ*H*_sub_, or the Gibbs free energy of sublimation, entropy of sublimation, and enthalpy of sublimation, respectively, will be calculated using methods in thermogravimetric analysis (TGA). By understanding these values, the approximate retention times of the rare earth species can be predicted using kinetic models, or by thermodynamic methods such as those reported by Eichler et al. [7].

There is interest in the determination of rate-dependent parameters of non-isothermal sublimation by analysis of TG (thermogravimetric) curves and differential TG (DTG) curves. Most commonly found methods of analysis of such curves include the Horowitz-Metzger (HM), Coats-Redfern (CR) and Freeman-Carroll (FC) methods. Each uses a different approach in relating mass loss as a function of temperature change. Each equation can be graphed linearly, where the slope of the line can be used to solve for the sublimation enthalpy and the *y*-intercept used to find a pre-exponential factor, *Z*, used in the calculation of the sublimation entropy. The resulting equations are shown below, while their derivations are omitted for brevity [17–19].

### Horowitz-Metzger equation

The Horowitz-Metzger equation is found via an integral method (Eq. 1):

1 $$\ln \left[ {\frac{{(1 - (C_{\text{HM}} )^{1 - n} )}}{(1 - n)}} \right] = \frac{{E^{*} \theta }}{{{\text{RT}}_{s}^{2} }};\quad C_{\text{HM}} = \frac{{w - w_{\infty } }}{{w_{0} - w_{\infty } }};\quad \theta = T - T_{s}$$

where E^*^ is the activation energy and found from the slope, *n* is the sublimation reaction order specific to the compound, *R* is the ideal gas constant, and *T*_s_ is the sublimation temperature, or the temperature at the peak on the DTG curve. The weight of the sample is represented as *w*, while the weight at beginning and at completion are represented as *w*_0_ and *w*_∞_, respectively. The pre-exponential factor, *Z*, is calculated using Eq. 2.

2 $$Z = \frac{{E^{*} \beta }}{{{\text{RT}}_{s}^{2} }}\exp \left( {\frac{{E^{*} }}{{{\text{RT}}_{s} }}} \right)$$

### Coats-Redfern equation

The Coats-Redfern equation is found via an integral method using Eq. 3.

3 $$\log \left[ {\frac{{(1 - (1 - C_{\text{CR}} )^{1 - n} )}}{{(1 - n)T^{2} }}} \right] = \log \left[ {\frac{\text{ZR}}{{\beta E^{*} }}\left( {1 - \frac{{2{\text{RT}}_{s} }}{{E^{*} }}} \right)} \right] - \frac{{E^{*} }}{{2.3.3{\text{RT}}}};\quad C_{\text{CR}} = \frac{{w_{0} - w}}{{w_{0} - w_{\infty } }}$$

where the variables are as above, *Z* is the pre-exponential factor in s^−1^ and can be found from the *y*-intercept, and *β* is the heating rate in °C/s.

### Freeman-Carroll equation

The Freeman-Carroll equation is found via a differential method using Eq. 4.

4 $$\ln \left[ {\frac{{{\text{d}}C_{\text{FC}} }}{{{\text{d}}T}}/(1 - C_{\text{FC}} )^{n} } \right] = \ln \left[ {\frac{z}{\beta }} \right] - \frac{{E^{*} }}{\text{RT}};\quad C_{\text{FC}} = C_{\text{CR}} = \frac{{w_{0} - w}}{{w_{0} - w_{\infty } }}$$

where the variables are as above and the derivative of *C*_FC_ with respect to temperature is needed.

Regardless of method, the sublimation thermodynamic parameters can be found using Eqs. 5–7.

5-7 $$\Delta H_{\text{sub}} = E^{*} - {\text{RT}}_{s} \quad {\text{and}}\quad \Delta S_{\text{sub}} = R\ln \left( {\frac{hz}{{kT_{s} }}} \right)\quad {\text{and}}\quad \Delta G_{\text{sub}} = \Delta H_{\text{sub}} - T_{s} \Delta S_{\text{sub}} \,$$

where the variables are as above, *h* is Planck’s constant, and *k* is Boltzmann’s constant.

## Experimental

### Synthesis: hfac, hfod, and hdpm Complexes

All reagents and solvents were used from commercial sources and used without further purification. The synthesis of the complexes followed that reported in the literature [9–16]. The rare earth oxides were dissovled in conc. HCl and were allowed to evaporate to produce LnCl_3_, where Ln represents any element in the rare earth series La–Lu, excluding Ce and Pm. The 1,1,1,5,5,5-hexafluoroacetalacetone (hfac) was treated with NH_4_OH to form a white precipiate, as was the 6,6,7,7,8,8,8-heptafluoro-2,2-dimethyl-3,5-octanedione (fod). The hfac or fod were then combined in a 4:1 molar ratio with the LnCl_3_ to produce the Ln[hfac]_4_ or Ln[fod]_3_. The 2,2,6,6-tetramethyl-3,5-heptanedione (dpm) was combined under Ar with a 4 M NaOH solution, which was stirred vigourously. The LnCl_3_ was added in 1:4 molar ratio as an aqueous solution to the reaction vessel and allowed to vacuum reflux overnight to prduce Ln[dpm]_3_. The products were all collected via vacuum filtration.

### Thermodynamic measurements

All TGA was performed on a Perkin Elmer Pyris 1 instrument. Samples of arbitrary mass between 3 and 6 mg were run under nitrogen, held at 105 °C for 5 min or until signal equilibrated within 0.005 °C, heated from 105 to 350 °C at 10 °C/min, then held at 350 °C for two additional minutes. This entails placing samples in high-temperature platinum (HT) pans with similar surface area, heating under desired temperature program, and repeating twice more for comparison.

Differential scanning calorimetry (DSC) is achieved via numerical differentiation of the raw TGA data curve with respect to temperature. A similar curve is utilized in the Freeman-Carroll method as described above. The analysis of the curves requires line-fitting, and is done so only over the region of sublimation, i.e., the entire peak of the DSC curve.

## Results and discussion

### Thermodynamics results

**Fig. 1:**
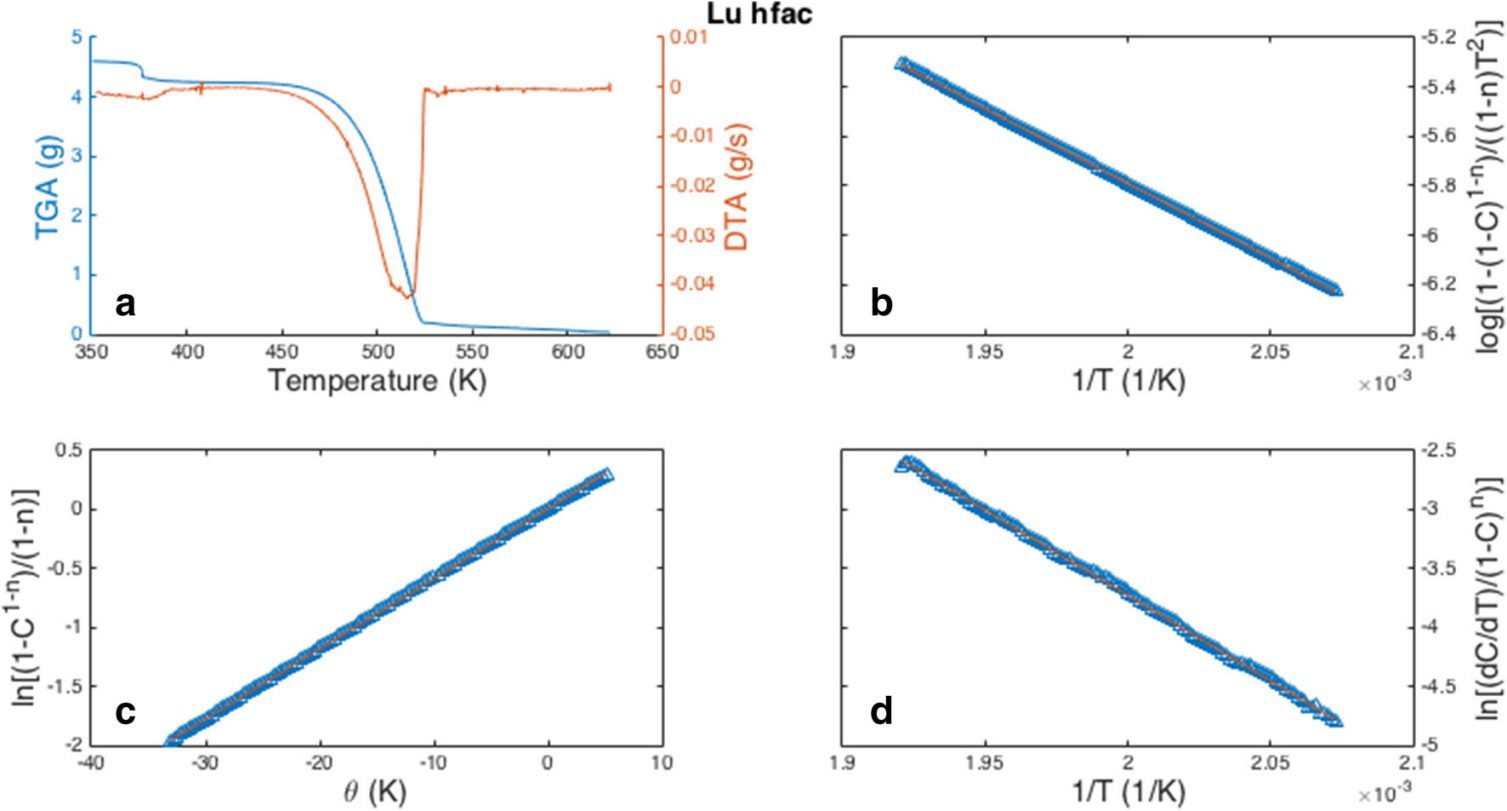
**a** The TGA/DSC data for the NH_4_ *·*Lu[hfac]_4_ compouds, **b** Coats-Redfern method, **c** Horowitz-Metzger method, and **d** Freeman Carroll method

The TGA/DSC results are presented in the following figures. A number of methods for analyzing the TGA/DSC data have been reported in the literature, and for this work the methods reported by Freeman-Carroll, Horowitz-Metzger, and Coats-Redfern have been applied [17–19]. The results for NH_4_·Lu[hfac]_4_ have been shown as example (Fig. 1a–d), with all of the hfac compounds having a similar curve shape, and the results given in Table 1. The loss of NH_4_^+^, or water, from the compounds is observed in the first region near 320 K. The Horowitz-Metzger and Coats-Redfern methods produce very similar plots and have similar results for their Δ*G*_sub_, Δ*S*_sub_, and Δ*H*_sub_. The Freeman-Carroll method provides a very different treatment of the data and has different results. The large difference in mass between 480 and 520 K is the region where sublimation of the compounds occurs.

**Table 1:** The complete thermodynamic parameters for the Ln[hfac], Ln[fod], and Ln[dpm] compounds

Comp.	Meth.	*T*_s_ (K)	Range (K)	Δ*S*_sub_ (kJ/mol K)	Δ*H*_sub_ (kJ/mol)	Δ*G*_sub_ (kJ/mol)
Dy hfac	CR	504	471–509	−0.1109	85.94	141.85
Dy hfac	HM			−0.0827	99.89	141.58
Dy hfac	FC			−0.1236	79.99	142.31
Dy hfod	CR	500	466–505	−0.0796	100.23	140.01
Dy hfod	HM			−0.0491	115.24	139.78
Dy hfod	FC			−0.0561	111.71	139.75
Er hfac	CR	484	451–489	−0.1445	66.76	136.75
Er hfac	HM			−0.1173	79.64	136.44
Er hfac	FC			−0.0624	105.53	135.75
Eu dpm	CR	557	524–562	−0.1059	98.46	157.46
Eu dpm	HM			−0.0787	113.42	157.25
Eu dpm	FC			−0.0961	103.85	157.37
Eu hfac	CR	488	455–493	−0.1533	63.34	138.18
Eu hfac	HM			−0.1264	76.07	137.76
Eu hfac	FC			−0.0077	132.84	136.62
Gd hfac	CR	488	455–493	−0.2243	30.80	140.24
Gd hfac	HM			−0.2019	41.53	140.04
Gd hfac	FC			0.0019	138.89	137.96
Ho dpm	CR	512	479–517	−0.0928	96.38	143.92
Ho dpm	HM			−0.0637	111.04	143.65
Ho dpm	FC			−0.0735	105.96	143.62
Ho hfac	CR	480	449–485	−0.0503	109.46	133.59
Ho hfac	HM			−0.0191	124.24	133.41
Ho hfac	FC			−0.0373	115.51	133.40
La hfac	CR	474	442–479	−0.2058	37.91	135.45
La hfac	HM			−0.1818	48.93	135.12
La hfac	FC			−0.0309	118.58	133.20
La hfod	CR	553	519–559	−0.1280	86.11	156.93
La hfod	HM			−0.1015	100.48	156.63
La hfod	FC			−0.2388	27.43	159.53
Lu hfac	CR	516	482–521	−0.0633	111.67	144.33
Lu hfac	HM			−0.0327	127.24	144.11
Lu hfac	FC			−0.0546	116.10	144.25
Nd hfac	CR	477	445–483	−0.2512	18.12	138.06
Nd hfac	HM			−0.2311	27.86	138.18
Nd hfac	FC			0.0025	136.91	135.72
Nd hfod	CR	560	527–565	−0.0743	116.49	158.11
Nd hfod	HM			−0.0443	132.74	157.56
Nd hfod	FC			0.0674	194.22	156.45
Pr dpm	CR	539	505–544	−0.0610	118.23	151.10
Pr dpm	HM			−0.0310	134.31	151.04
Pr dpm	FC			−0.1019	96.80	151.75
Pr hfac	CR	477	445–482	−0.1344	70.55	134.59
Pr hfac	HM			−0.1050	83.82	133.86
Pr hfac	FC			0.1323	194.71	131.66
Sm hfac	CR	465	434–470	−0.2451	20.10	134.06
Sm hfac	HM			−0.2244	29.71	134.05
Sm hfac	FC			0.0486	154.07	131.47
Sm hfod	CR	551	518–556	−0.1348	82.23	156.53
Sm hfod	HM			−0.1084	96.46	156.20
Sm hfod	FC			−0.0892	107.21	156.35
Tm dpm	CR	524	491–529	−0.1527	68.80	148.88
Tm dpm	HM			−0.1271	81.97	148.64
Tm dpm	FC			−0.1145	88.40	148.45

**Fig. 2:**
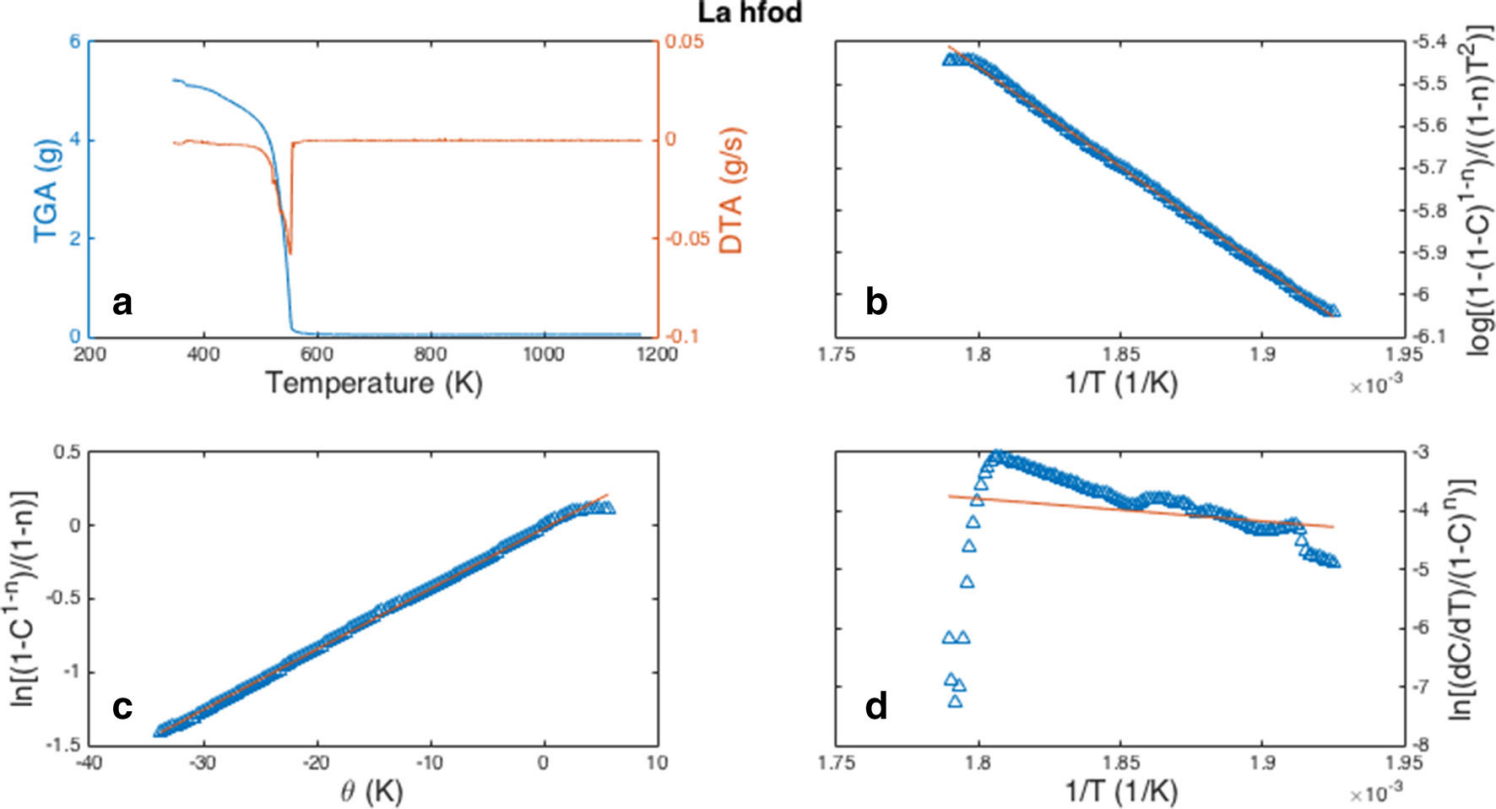
**a** The TGA/DSC data for the La[fod]_3_ *·*H_2_O compouds, **b** Coats-Redfern method, **c** Horowitz-Metzger method, **d** Freeman Carroll method

In a similar fashion, the Ln[fod] compounds were also analyzed via TGA/DSC, with La[fod]_3_·H_2_O shown as example (Fig. 2a–d). Like the previous analysis, the Horowitz-Metzger method and the Coats-Redfern methods remain similar in their treatment of the data. The Freeman-Carroll method, however does not fit the data nearly as well. The region between 380 and 400 K is likely where any additional water is removed from the complex, and the region between 550 and 600 K is where the fod compounds generally sublime.

**Fig. 3:**
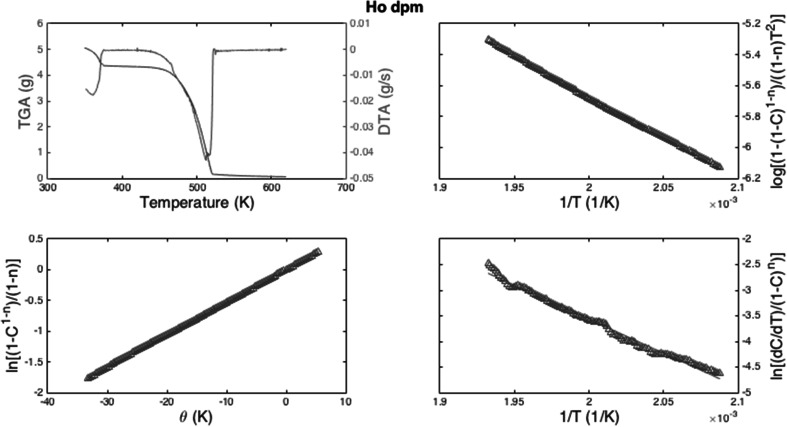
**a** The TGA/DSC data for the Ho[dpm]_3_ *·*H_2_O compouds, **b** Coats-Redfern method, **c** Horowitz-Metzger method, **d** Freeman Carroll method

The final compound analyzed was the Ln[dpm] series of complexes, where the Ho[dpm]_3_·H_2_O is depicted in Fig. 3 as example. Like the hfac data, the dpm samples were very similar in the curve shape of the data across the series, and the full results are compiled in Table 1. Yet similar to the hfod samples, in the region between 380 and 400 K most of the water is removed, and the region between 480 and 520 K is the point at which the compounds sublime. Similarly, the Horowitz-Metzger and Coats-Redfern methods provide similar treatments to the data and similar results, while the Freeman–Carroll method gives a very different result as shown in Table 1.

The complete results from all of the TGA/DSC methods are presented in Table 1. The ionic radius of the method ion is given in angstroms, the T_s_ is the temperature of sublimation, the range is the region of change (in K), the Δ*E* is the energy of activation for the process (in J), the A or Z factor is a correction factor corresponds the method used to analyze the compound. HM corresponds to the Horowitz-Metzger, CR corresponds to the Coats-Redfern method, and FC corresponds to the Freeman-Carroll method.

**Fig. 4:**
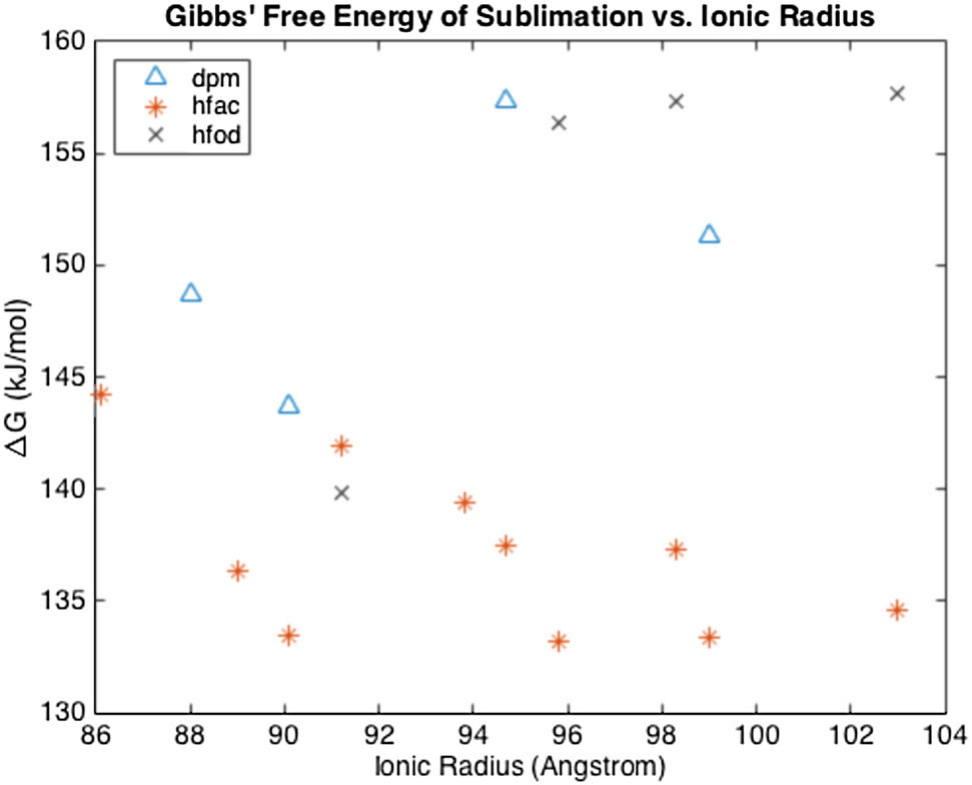
The average (of the HM and CR methods) Gibbs’ free energy of sublimation (ordinate) is plotted as a function of the ionic radius (abscissa)

The results of the table are shown graphically in Fig. 4, where the average (of the HM and CR methods) Gibbs’ free energy of sublimation (abscissa) is plotted as a function of the ionic radius of the metal compounds.

The Ln[hfac] compounds trend towards lower values of Δ*G*, while the Ln[fod] compounds trend towards larger values as the ionic radius increases. The Ln[dpm] compounds do not have an apparent trend as a function of atomic radius.

### Prediction of retention times

The thermodynamic data can be used to predict the retention times in thermochromatographic experiments as noted by Eichler et al. [7]. The retention times can be determined using Eq. 8.

8 $$t_{r} = \frac{{LT_{0} \phi }}{{\bar{V}_{0} T_{\text{iso}} }} \times \left( {1 + \frac{s}{v} \times \frac{V}{100A} \times \exp \left( { - \frac{{\Delta H_{\text{ads}}^{0} }}{{RT_{\text{iso}} }}} \right) \times \exp \left( {\frac{{\Delta S_{\text{ads}}^{0} }}{R}} \right)} \right)$$

where *L* is the length of the column, *T*_0_ is standard temperature 298.15 K, *ϕ* is the free open cross-sectional area of the column, *V*_0_-*bar* is the carrier gas flow at STP (standard temperature and pressure), *T*_iso_ is the isothermal column temperature, *s* is the open surface of column per 1 m column length, *v* is the open volume of the column per 1 m column length, *V* is the inner volume of the column, *A* is the inner surface per 1 m of column length, and *R* is the ideal gas constant. The entropy of adsorption can be calculated from the previous equation, while the enthalpy of adsorption can be found using Eq. 9.

9 $$- \Delta H_{\text{ads}}^{0} = \left( {2.9 \pm 16} \right) + (.73 \pm .1) \times \Delta H_{\text{subl}}^{0}$$

where the enthalpy of sublimation was taken from the thermodynamic models mentioned previously. The Coats-Redfern and the Horowitz-Metzger methods were used for calculation of the parameter, while the Freeman-Carroll was not used due to the inconsistent nature of parameter values obtained from the method. The calculation of $\Delta S_{\text{ads}}^{0}$ can be done using Eq. 10.

10 $$\Delta S_{\text{ads}}^{0} = R\left( {\ln \left( {\frac{100A}{{V \times v_{b} }}} \right) \times \sqrt {\frac{R \times T}{{2 \times \pi \times M_{\text{a}} }} + \frac{1}{2}} } \right)$$

where the entropy of adsorption is related to *R*, the ideal gas constant, the area of the column, *A*, the volume of the column, *V*, the phonon frequency of the column material (e.g., quartz, etc.), *ν*_*b*_, the temperature, *T*, and the mass of the adsorbing material, *M*_a_. The approximate retention times using a thermochromatography unit fitted with a 30 m SiO_2_ column operating at 150 °C, with a flow rate of 0.8 cm/s and an inner diameter of 0.5 mm, were approximated and tabulated in Table 2.

**Table 2:** The calculated retention times for selected Ln[hfac], Ln[fod], and Ln[dpm] compounds

	Ho[dpm]	La[fod]	Lu[hfac]
MW (g/mol)	734.37	1045.47	1025.24
Δ*H*_subl_ (kJ/mol)	103.7	93.3	119.5
Δ*H*_ads_ (kJ/mol)	−78.6	−71.0	−90.1
Δ*S*_ads_ (kJ/mol K)	−0.142	−0.119	−0.120
*t*_r_ (s)	1.496E+05	2.965E+05	5.789E+07

## Conclusions

This work represents the first reports of the thermodynamic parameters of the β-diketonate complexes of Ln[hfac], Ln[hfod], and Ln[hdpm]. The thermodynamics have been used to begin to establish the feasibility of using thermochromatography as a technique to perform rapid separation of fission products. Knowledge of the thermodynamics of these compounds can be beneficial in developing approximations of retention times. Initial approximations from the model are much longer than hypothesized, and in initial chromatographic experiments, the times observed are much shorter. The observed discrepancy is largely due to the approximations for a variety of the theoretical parameters of the model and the differences in chemistry between the lanthanide β-diketonates and the super heavy elements. Future work will involve the refinement of the model’s parameters and its operation, as well as the introduction and detection of the aforementioned compounds on an isothermal chromatographic column.
